# Risk factors of fatigue among community-dwelling older adults in Bahir Dar, Northwest Ethiopia: a community-based cross-sectional study

**DOI:** 10.3389/fpubh.2024.1491287

**Published:** 2024-11-20

**Authors:** Belayneh Addis Mekuria, Molla Fentanew, Yeshambel Ejigu Anteneh, Jemal Suleman, Yihalem Belet, Kefale Getie, Haimanot Melese, Fiseha Sefiwu Zinabu, Mihret Dejen Takele, Kassahun Cherkos, Assefa Gebeyehu Muluneh, Gashaw Jember Belay

**Affiliations:** ^1^Department of Physiotherapy, College of Medicine and Health Sciences, School of Medicine, Bahir Dar University, Bahir Dar, Ethiopia; ^2^Department of Physiotherapy, College of Medicine and Health Sciences, School of Medicine, University of Gondar, Gondar, Ethiopia

**Keywords:** fatigue, prevalence, older adults, factors, Ethiopia, associated factors

## Abstract

**Background:**

Fatigue is defined as subjective fatigue and a decline in physical and mental activity that does not improve with rest. Fatigue among older adults could lead to future comorbidity, mortality, decreased social interaction, greater strain on families, decreased productivity, and a higher need for hospitalization and rehabilitation. However, no studies have been conducted in Africa, particularly in Ethiopia. Therefore, this study aimed to evaluate the prevalence and factors of fatigue among older adults.

**Methods:**

A community-based cross-sectional study of 605 older adults was carried out using a single-stage cluster sampling technique. The Chalder Fatigue Scale (CFS) was used to assess fatigue, and data were collected through an interview. The collected data were coded, cleaned, and entered into EpiData version 4.6 and exported to SPSS Version 25 for analysis. Bivariate and multivariate logistic regression analyses were performed. Variables in the final multivariate logistic regression model with a 95% confidence interval (CI) and a *p*-value of 0.05 were considered statistically significant.

**Results:**

The prevalence of fatigue among older adults was 37.9% (95% CI, 34–41.90). Significant risk factors included older age [adjusted odds ratio (AOR) = 6.13, CI = 3.25–11.58], the presence of two or more comorbidities (AOR = 5.68, CI = 2.97–10.83), physical inactivity (AOR = 3.33, CI = 1.56–7.12), poor social support (AOR = 2.83, CI = 1.61–4.96), insomnia (AOR = 5.48, CI = 3.38–8.88), and depression (AOR = 2.65, CI = 1.60–4.36).

**Conclusion:**

The prevalence of fatigue among older adults was noticeable, and it was summarized as a public health issue among older adults in the study area. Our study findings revealed that older age, the presence of comorbidities, physical inactivity, poor social support, insomnia, and depression were all risk factors for fatigue among community-dwelling older adults.

## Background

Fatigue is defined as a subjective state of overwhelmingly prolonged debilitation and diminished capacity for physical and mental endeavors that is hardly alleviated by rest (1). Physical fatigue can be defined as an impairment in the ability to apply force or power, regardless of whether the task can still be performed successfully. In contrast, ramification is a type of fatigue that results in mental fatigue (2). Rest typically relieves fatigue in healthy individuals, which is a natural episode after physical or mental effort (3, 4).

The World Health Survey demonstrated that the burden of fatigue among community-dwelling individuals aged >60 years ranged from 27% to 50% (5, 6). Statistics from the Global Health Survey showed that among studies conducted in 10 European nations among community-dwelling older adults aged 65 years or older, the rates of fatigue were as high as 55% (7).

Fatigue has significant implications for human health; it can signal impending illness and mortality and undermine capacity, social cohesion, and motivation, despite its debilitating nature (8–11). Understanding the factors related to fatigue and implementing effective intervention schemes could help prevent fatigue, mitigate the process of aging, and reduce the odds of symptoms persisting after hospital discharge (12). Fatigue is a common symptom among older adults, yet it is often overlooked, with many patients and healthcare professionals assuming it to be an innate evolution of aging (13).

Factors contributing to episodes of fatigue or its progression are diverse and include socio-demographic, clinical, lifestyle-related, and biological factors. Studies conducted among older adults have indicated that the risk factors for fatigue include older age, being unmarried or widowed, having above and below normal body mass index (BMI), being female, low economic status, lower educational level, a history of hospitalization in the past year, comorbidities, depression, insomnia, poor social support, living alone, activities of daily living (ADL) dependency, physically inactivity, and cigarette smoking (14–28).

As evidence has demonstrated, there are no published studies on the prevalence and associated factors of fatigue among older adults in sub-Saharan Africa, including Ethiopia. Therefore, this study aimed to assess the prevalence of fatigue and identify the associated risk factors among community-dwelling older adults in Bahir Dar, Northwest Ethiopia.

## Methods

### Study design and setting

A community-based cross-sectional study was conducted from April to June 2023. The study was conducted in Bahir Dar, Northwest Ethiopia. The source population included all community-dwelling older adults aged 60 years or older. The study population consisted of older adults aged 60 years or older from selected kebeles during the study period. Those older adults who permanently resided for ≥6 months in the selected kebeles were included. The included study participants were both disease-free and had a formal diagnosis. Older adults who could not communicate, were critically ill, or had experienced an acute stroke were excluded.

### Sample size determination

The sample size was determined using a single population proportion formula, with the assumptions: a 50% population proportion, a 95% confidence interval (CI), a marginal error of 5%, and a 10% non-response rate.


$$\begin{array}{r} n\;=\frac{{\left(Z_{\alpha /2}\right)}^{2}p\;\left(1-p\right)}{d^{2}}\;\\ N\;=1.962\times 0.5\times 0.5/0.052\\ =3.8416\times 0.25/0.0025\;=\;384.37\sim 385.\; \end{array}$$

By considering a design effect of 1.5 and a 10% non-response rate multiplier, the minimum required sample size was 536. However, because of the effect of cluster sampling, a total of 651 older adults participated in the study.

### Sampling technique and procedure

**Figure 1:**
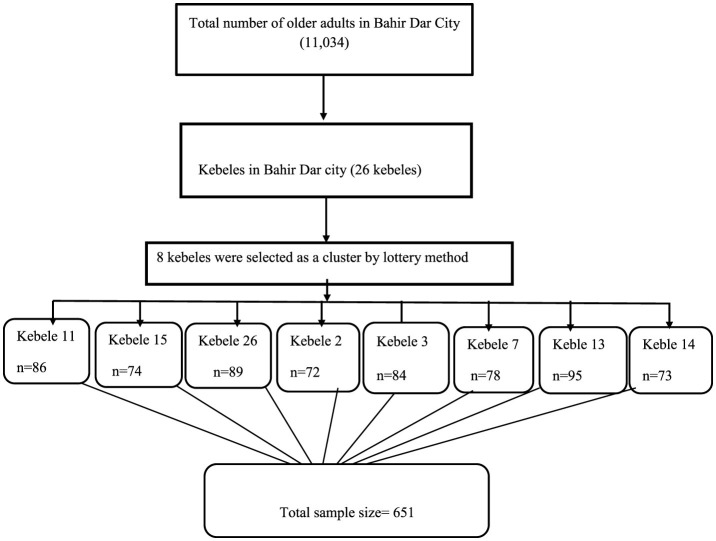
Schematic representation of the sampling procedure among the community-dwelling older adults in Bahir Dar, Ethiopia, 2023.

We recruited study participants from Bahir Dar, which has a population of 11,034 older adults across 26 kebeles. Eight kebeles were selected using the lottery method, and participants from selected kebeles were allocated proportionally, with each kebele contributing between 72 and 95 participants. The study participants were both disease-free and those with a formal diagnosis. A single-stage cluster sampling technique was used to select the study participants. All eligible older adults within the selected cluster were interviewed in their households (Figure 1).

### Data collection procedures

Ethical clearance was obtained from the ethical review committee of the University of Gondar, College of Medicine and Health Sciences, and informed consent was obtained from the study participants. After the participants provided informed consent, five trained healthcare professionals conducted a face-to-face interview using predefined and pretested structured questionnaires. The questionnaires had five components: sociodemographic characteristics, health-related factors, psychosocial factors, behavioral-related factors, and the Chalder Fatigue Scale (CFS).

The other in-depth sections of the tool were developed based on previous articles, and the questionnaires were modified according to all the factors that were related to the study objectives. The English version of the questionnaires was translated into the Amharic language for data collection and then back into English for analysis to ensure consistency.

### Data collection instruments and operational definitions

#### Fatigue

Individuals with a CFS score of ≥4 are considered to be fatigued. The CFS is an easy-to-understand, brief, and validated scale for assessing fatigue in older adults. It provides reliable and valid fatigue measurements, with α = 85%. It has been widely used because of its reliability and validity in assessing fatigue in both general and clinical populations. It consists of 11 items, with total scores ranging from 0 to 11. Each item has a score of either 0 or 1. The older participants who scored >4 were considered to be fatigued (29, 30).

#### ADL dependency

It was measured using the Katz Index of Independence in Activities of Daily Living, with α = 84%. Individuals with a score of ≤ 5 are considered to have ADL dependence. It is commonly used to assess the functional status of older adults. The index ranks the adequacy of performance in six activities of daily living: bathing, dressing, toileting, transferring, continence, and feeding. The scores are categorized as “yes = 1” and “no = 0” for independence in each of the six activities, with the attainable score ranging from 0 to 6. A score of 6/6 indicates full function, 4/6 indicates moderate impairment, and 2/6 or less indicates severe functional impairment. The study participants who scored ≤ 5 were considered to have ADL dependence (31).

#### Depression

It was measured using the Geriatric Depression Scale (GDS), with α = 92%; Individuals with a score ≥5 are considered to be depressed. The GDS is used to screen for depressive symptoms, with the attainable score ranging from 0 to 15. Participants respond with “yes” or “no” to the 15 statements that describe either a positive or negative emotion (32).

#### Older adults

A person whose age is ≥60 years is referred to as an older adult (19, 27, 33).

#### Body mass index (BMI)

Weight in kilograms divided by the square of height in meters (kg/m^2^) with the following categories: underweight (< 18.50), normal weight (18.50–24.99), overweight (≥25.00–29.9), and obese (30.0 and above) (34).

#### Physical activity

It is defined as any moderate-intensity exercise (such as walking, cycling, sports, or planned exercise and strength training) performed for at least 150 min per week (35).

#### Smoker

Smokers were defined as adults who had smoked at least 100 cigarettes in their lifetime and currently smoke every day. Former smokers were adults who had smoked at least 100 cigarettes in their lifetime but had quit smoking at the time of the interview (36).

#### Insomnia

It was assessed using the Insomnia Severity Index (ISI), with α =79%. The index consists of seven items, with scores ranging from 0 to 28, and a score >15 indicates moderate to severe intensity (37).

#### Social support

It was assessed using the Oslo Social Support Scale (OSSS-3), with α = 64%. The total score ranges from 3 to 14, with 3–8 points indicating poor social support, 9–11 points indicating moderate social support, and 12–14 points indicating strong social support (38).

#### Kebeles

A kebele is the name of a place used for zoning purposes in administrative activities. For example, in Ethiopia, the administrative hierarchy in the city is as follows: city–sub-city–woreda–kebele. Therefore, a kebele is at the lowest level in the administrative structure.

### Data quality assurance and management

To ensure the quality of the data, the data collectors and supervisors were trained by the principal investigators for 1 day before the start of the actual data collection. The training covered how to approach the participants, the objective of the study, and ethical issues. The supervisor ensured the completeness and consistency of the data. The questionnaires were pretested in Merawi before the actual data collection to assess the accuracy of the responses, language clarity, and the appropriateness of the questions. Pre-testing was performed on 5% of the total sample. It was carried out before the actual data collection period on a population with similar characteristics to the study population but not part of the actual study group. For the present study, the necessary changes were made based on the findings of the pretest.

### Statistical analyses

The collected data were edited, coded, cleaned, and subsequently entered into EpiData version 4.6. After export, the data were analyzed using SPSS version 25. In the bivariate logistic regression analysis, variables with a *p*-value < 0.25 were considered potential candidates for the final multivariable logistic regression analyses. Variables with a *p*-value of < 0.05 at a 95% confidence interval (CI) were considered statistically significant, and their odds ratio (OR) was used to interpret the findings of the final model.

## Results

### Sociodemographic characteristics of the study participants

Overall, 605 older adults participated in this study, with a 93% response rate. Among the total respondents, the majority [325 (53.7%)] were female (Table 1).

**Table 1:** Sociodemographic characteristics of the community-dwelling older adults in Bahir Dar, Northwest, Ethiopia, 2023 (*n* = 605).

**Variables**	**Categories**	**Frequency (%)**	**Fatigue status**	
			**Yes (%)**	**No (%)**
Sex	Male	280 (46.3)	90 (32.1)	190 (67.9)
	Female	325 (53.7)	139 (42.8)	186 (57.2)
Age in years	60–64	147 (24.3)	32 (21.8)	115 (78.2)
	65–69	226 (37.4)	46 (20.4)	180 (79.6)
	≥70	232 (38.3)	151 (65.1)	81 (34.9)
Educational status	Cannot read and write	80 (13.2)	56 (70)	24 (30)
	Primary school (1–8)	220 (36.4)	70 (31.8)	150 (68.2)
	Secondary school (9–12)	173 (28.6)	54 (31.2)	119 (68.8)
	College and above	132 (21.8)	49 (37.1)	83 (62.9)
Marital status	Married	242 (40)	34 (14)	208 (86)
	Divorced	158 (26.1)	67 (42.4)	91 (57.6)
	Windowed	205 (33.9)	128 (62.4)	77 (37.6)
Living arrangement	Living alone	56 (9.3)	13 (23.2)	43 (76.8)
	Living with spouse	262 (43.3)	120 (41.8)	167 (58.2)
	Living with children	287 (47.4)	96 (36.6)	166 (63.4)
Income status	< 1,500	73 (12.1)	20 (27.4)	53 (72.6)
	1,500–3,500	88 (14.5)	39 (44.3)	49 (55.7)
	>3,500	444 (73.4)	170 (38.9)	274 (61.1)
Body mass index	Normal weight	454 (75)	160 (35.2)	294 (64.8)
	Underweight	69 (11.4)	26 (37.7)	43 (62.3)
	Overweight	82 (13.6)	43 (52.4)	39 (47.6)

### Psychosocial and health-related factors of the participants

According to the GDS screening, more than one-fourth of the participants had depressive symptoms. In addition, 164 (27.1%) had poor social support, 266 (44%) had insomnia, 108 (17.9%) had two or more comorbidities, 21 (3.5%) had a history of hospitalization, and 509 (84.1%) were physically inactive (Table 2).

**Table 2:** Psychosocial, health, and behavioral characteristics of the older adults in Bahir Dar, Northwest Ethiopia, 2023 (*n* = 605).

**Variables**	**Categories**	**Frequency (%)**	**Fatigue status**	
			**Yes (%)**	**No (%)**
Depression	Yes	181 (29.9)	118 (65.2)	63 (34.8)
	No	424 (70.1)	111 (26.2)	313 (73.8)
Insomnia	Yes	266 (44)	173 (65)	93 (35)
	No	339 (56)	56 (16.5)	283 (83.5)
Social support	Poor	164 (27.1)	110 (67.1)	54 (32.9)
	Moderate	127 (21.0)	39 (30.7)	88 (69.3)
	Strong	314 (51.9)	80 (25.5)	234 (74.5)
Comorbidity	No	497 (82.1)	83 (76.9)	25 (23.1)
	Yes	108 (17.9)	146 (29.4)	351 (70)
Hospitalization	No	584 (96.5)	11 (52.4)	10 (47.6)
	Yes	21 (3.5)	218 (37.3)	366 (62.7)
ADL dependency	No	490 (81)	190 (38.8)	300 (61.2)
	Yes	115 (19)	39 (33.9)	76 (66.1)
Physical activity	No	501 (82.8)	213 (42.5)	288 (57.5)
	Yes	104 (17.2)	16 (15.4)	88 (84.6)
Smoking	Yes	39 (6.4)	13 (33.3)	26 (66.7)
	No	566 (93.6)	216 (38.2)	350 (61.8)

### The prevalence of fatigue among study participants

**Figure 2:**
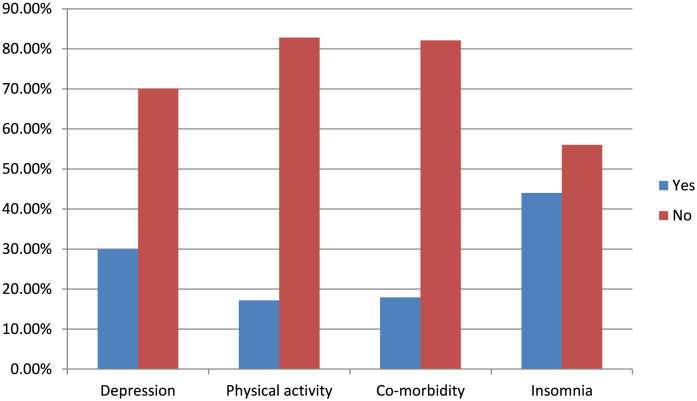
Distribution of fatigue in relation to depression, physical activity, comorbidity, and insomnia among the community-dwelling older adults in Bahir Dar, Northwest Ethiopia, 2023 (*n* = 605).

The overall prevalence of fatigue among older adults was 37.9% (95% CI, 34.00–41.90). Among them, 60.7% were female, 65.1% were 70 years old or older, 62.4% were widowed, and 52.4% were overweight (Tables 1, 2 and Figure 2).

### Factors associated with fatigue among older adults

We entered the independent variables into a bivariate logistic regression, and those with a *p*-value < 0.25 were included in a multivariable logistic regression to control for potential confounding factors. The multicollinearity of the variables was assessed using the variance inflation factor (VIF), and the values for all variables were < 10. The Hosmer–Lemeshow test yielded a value of 0.61. In the final model, the variables such as age 70 years or older, physical inactivity, having two or more comorbidities, depression, poor social support, and insomnia were significantly associated with fatigue (95% CI, *p* < 0.05).

Participants aged 70 years or older were 6 times more likely to develop fatigue than those aged 60–64 years [adjusted odds ratio (AOR) = 6.13, CI = 3.25–11.58]. Furthermore, older adults with two or more comorbidities were 5.6 times more likely to develop fatigue than those with no or one comorbidity (AOR = 5.68; CI = 2.97–10.83). Similarly, the older adults with insomnia were 5.4 times more likely to develop fatigue than those without insomnia (AOR = 5.48; CI = 3.38–8.88). Moreover, the older adults with depressive symptoms were 2.6 times more likely to develop fatigue than those without depressive symptoms (AOR = 2.65; CI = 1.60–4.36). The older adults with poor social support were 2.8 times more likely to experience fatigue than those with strong social support (AOR = 2.83; CI = 1.61–4.96). In addition, the older adults who were physically inactive were 3.3 times more likely to develop fatigue than those who were physically active (engaged in exercise; AOR = 3.33; CI = 1.56–7.12; Table 3).

**Table 3:** Factors associated with fatigue according to the bivariate and multivariate logistic regression analyses of the study participants among community-dwelling older adults in Bahir Dar, Northwest Ethiopia 2023 (*n* = 605).

**Variables**	**Categories**	**COR (95%)**	**AOR (95%)**
Age in years	60–64	1	1
	65–69	0.91 (0.55–1.52)	1.03 (0.53–1.99)
	>70	**6.70 (4.16–10.78)**	**6.13 (3.25–11.58)** ^**^
Sex	Male	**1**	**1**
	Female	1.57 (1.13–2.20)	0.95 (0.57–1.56)
Educational level	Unable to read and write	3.95 (2.18–7.16)	1.43 (0.52–2.70)
	Primary school	0.79 (0.50–1.24)	0.56 (0.29–1.06)
	Secondary school	0.77 (0.48–1.23)	0.63 (0.42–1.64)
	College and above	**1**	**1**
Physical activity	No	**4.07 (2.32–7.13)**	**3.33 (1.56–7.12)** ^**^
	Yes	**1**	**1**
Comorbidity	No	**1**	**1**
	Yes	**7.98 (4.90–12.99)**	**5.68 (2.97–10.80)** ^**^
Depression	No	**1**	**1**
	Yes	**5.28 (3.63–7.68)**	**2.65 (1.60–4.36)** ^**^
Body mass index	Normal weight	**1**	**1**
	Underweight	1.11 (0.65–1.87)	1.02 (0.58–2.59)
	Overweight	2.02 (1.26–3.25)	1.52 (0.91–3.19)
Insomnia	Yes	**9.40 (6.42–13.70)**	**5.48 (3.38–8.88)** ^**^
	No	1	1
Social support	Poor	**5.95 (3.94–9.00)**	**2.8 (1.61–4.96)** ^**^
	Moderate	1.29 (0.82–2.04)	0.68 (0.35–1.30)
	Strong	1	1

1, reference category; CI, confidence interval.

^**^Highly statistically significant at p < 0.001. Bold value to indicate significant variables in multivariate logistic regression.

## Discussion

This study aimed to assess the prevalence of fatigue and its associated factors among community-dwelling older adults in Bahir Dar, Northwest Ethiopia, in 2023. The overall prevalence of fatigue was 37.9% (95% CI, 34.00–41.90). This finding indicated that more than one-fourth of the study participants are experiencing fatigue.

This finding is consistent with that of a study conducted in Turkey (40.3%). This may be due to the use of similar study designs, data collection methods, study settings, and participant age ranges. Most of the participants in the study conducted in Turkey and our group were independent and capable of performing daily tasks. The majority of the participants did not have comorbidities. Furthermore, the majority of the participants who reported fatigue were aged 70 years or older, inactive, and female (16).

The current study revealed a higher prevalence of fatigue compared to a study conducted in Canada (27%). This difference may be due to factors related to study design, study setting, health care, and inclusion and exclusion criteria. The study conducted in Canada was a prospective cohort study, with participants recruited from hospitals, and follow-up was conducted for 12 months. As a result, the participants might have been more aware of fatigue; appropriate treatment and preventive measures were provided. However, in our study, a cross-sectional design was used, and the participants were recruited from the community. Therefore, they might not have been unaware of fatigue and its preventive measures. Moreover, the study conducted in Canada excluded participants with schizophrenia, cognitive impairment, or low English literacy, conditions that may be associated with fatigue. In contrast, our study did not exclude these participants (26).

The present study reported a lower prevalence of fatigue compared to studies conducted in China (47.1%), Jordan (57%), South Korea (48.8%) (22), and Italy (44%). This discrepancy may be due to differences in study design, study setting, participants' utilization of health services, and the inclusion of different age groups. In the study conducted in China, the participants were recruited from hospitals and rural areas, and a self-administered data collection method was used. In contrast, our study recruited participants from the community and urban areas, and the data were collected using the face-to-face interview technique. Furthermore, the high level of fatigue among older adults in Jordan could be attributed to several factors: the participants were recruited from a health center, the use of the fatigue severity scale, and the fact that the majority of the participants had comorbidities. However, in our study, the participants were recruited from the community, the Chalder Fatigue Scale was used, and the majority of the respondents did not have comorbidities. In contrast to our study, the study conducted in South Korea used a convenience sampling technique to select participants from health services. In addition, 65% of the participants had at least two comorbidities, and the study focused on older adults aged 65 years or older. On the other hand, in our study, the participants were recruited through cluster sampling and from the community, and the majority of our study participants did not report comorbidities. In contrast to our study, the study conducted in Italy included participants aged 65–102 years, which might have increased the number of fatigue reports, and the data were collected using a self-administered technique. However, in our study, the participants' ages ranged from 60 to 89 years, and recruitment was performed using the cluster sampling method (14, 19, 20).

This study revealed that the participants aged 70 years or older were six times more likely to develop fatigue than those aged 60–64 years. The possible reason for this could be physiological changes associated with aging, as well as interactions between specific systems, such as inflammation and endocrine dysregulation, that increase the risk of fatigue. Generally, with aging, people experience reduced energy levels, a decline in physiological functions, and an increased risk of disease, all of which may further contribute to this phenomenon (12, 39). The results of this study are in agreement with those of studies conducted in China, Turkey, Italy, Israel, and Jordan (14–17, 19). Moreover, a systematic review conducted in the USA in 2020 on older individuals showed that advanced age increases the risk of fatigue (40).

This study showed that older adults with two or more comorbidities were 5.6 times more likely to experience fatigue than those with no or one comorbidity. This may be due to the accumulation of medical illnesses and other deficits at advanced ages, which predisposes individuals to adverse health outcomes such as physical inactivity, depressive symptoms, disability, prolonged hospital stays, complex pharmacological regimens, and increased vulnerability to fatigue (41, 42). Studies conducted in China, Italy, Jordan, and Israel (14, 17, 19, 20) support the current study's findings.

This study revealed that older adults with depressive symptoms were 2.6 times more likely to experience fatigue than those with no depressive symptoms. The possible reasons for this could be that depression and fatigue are linked in several ways: depression may directly cause fatigue, and chronic fatigue, in turn, may lead to depression (43). Depression also has indirect effects on sleep, diet, and physical activity (44). Consequently, the loss of muscle mass, strength, and exercise tolerance leads to an increase in cytokines, which is closely linked to the onset of fatigue (45, 46). This finding of the study is consistent with those of studies conducted in China, Pennsylvania, Jordan, South Korea, and Israel (17–20, 22).

This study reported that older adults who were physically inactive were 3.3 times more likely to experience fatigue than those who were physically active. A possible explanation could be that physical activity can reduce the risk of moderate to severe functional limitations in midlife and older adults, and it helps regulate metabolites and heat, which affect the stability of the internal environment (47, 48). This result is in agreement with studies conducted in Israel, Turkey, and Jordan (16, 17, 19).

The current study revealed that older adults with inadequate social support were 2.8 times more likely to experience fatigue compared to their counterparts with strong social support. A possible reason for this could be that poor social support is linked to depression and loneliness, both of which have been shown to alter brain function and increase the risk of alcohol use, cardiovascular diseases, and high blood pressure (49, 50). The study conducted in Jordan (19) supports the study's finding.

This study revealed that older adults with insomnia were 5.5 times more likely to experience fatigue compared to those without insomnia. A possible explanation could be that insomnia may cause both mental and physical fatigue, leading to an inability to accomplish tasks, a total lack of energy, and feelings of being overwhelmed. This can affect safety and make everyday life feel completely unmanageable (51, 52). Studies conducted in China, the USA, and Israel (17, 20, 23) support this study's findings.

### Strengths and limitations of the study

Our study's strengths include the collection of relevant data on fatigue status and the identification of associated factors using an internationally validated tool for the subjective assessment of physical and mental fatigue. Furthermore, we used a community-based study design and more than four outcome-measuring instruments.

The study's possible drawbacks include the use of cluster sampling and interview methods, which might have resulted in fewer responses to sensitive topics. In addition, due to the cross-sectional nature of the study design, causal relationships could not be firmly established.

## Conclusion

The prevalence of fatigue among older adults in Bahir Dar was notable and was identified as a public health issue in the study area. Age, multiple comorbidities, physical inactivity, poor social support, insomnia, and depression were associated with fatigue among older adults.

Our study findings suggest that the health bureau should emphasize reducing fatigue and promoting prevention strategies in health policy. Health professionals should provide continuous educational programs for older adults, especially those who have two or more comorbidities, depression, poor social support, insomnia, and are physically inactive, all of which require attention. The researchers should conduct more rigorous studies with a prospective study design to determine a nationally representative prevalence that includes rural older adults and assess the effectiveness of interventions.

## Data Availability

The original contributions presented in the study are included in the article/supplementary material, further inquiries can be directed to the corresponding authors.
